# Semi-occlusive dressing therapy versus surgical treatment in fingertip amputation injuries: a clinical study

**DOI:** 10.1007/s00068-022-02193-6

**Published:** 2022-12-10

**Authors:** Tatjana Pastor, Patricia Hermann, Luzian Haug, Boyko Gueorguiev, Torsten Pastor, Esther Vögelin

**Affiliations:** 1 Department of Hand Surgery, Bern University Hospital, University of Bern, Freiburgstrasse 18, 3010 Bern, Switzerland; 2 AO Research Institute Davos, Davos, Switzerland; 3 Department of Orthopaedic and Trauma Surgery, Lucerne Cantonal Hospital, Lucerne, Switzerland

**Keywords:** Fingertip amputation, Finger replantation, Hand surgery, Microsurgery, Semi-occlusive dressing

## Abstract

**Objectives:**

Treatment of fingertip amputations is subject of controversial debates. Recently, semi-occlusive dressings have increased in popularity in these injuries.

**Aims:**

To compare clinical outcomes of conservative semi-occlusive dressing therapy versus surgical treatment of fingertip amputations.

**Methods:**

Eighty-four patients with fingertip amputations were re-examined clinically after a mean follow-up of 28.1 months (range 9.6–46.2). Sixty-six patients (79%) were treated with semi-occlusive dressings (group 1) and 18 (21%) underwent surgery (group 2). Range of motion, grip strength, and two-point discrimination were measured at the final follow-up. Furthermore, VAS score, Quick-DASH score, subjective aesthetic outcome and loss of working days were obtained.

**Results:**

Group 1 demonstrated healing in all 66 patients (100%) while in Group 2 5 out of 18 patients (28%) failed to achieve healing after a mean of 17 days (range 2–38) due to graft necrosis. Group 1 showed significantly lower VAS scores and significantly lower loss of two-point discrimination compared to Group 2. Work absence was significantly shorter in Group 1 versus Group 2. Trophic changes in finger (46%) and nail (30%) were significantly lower in Group 1 compared to Group 2 (44% and 70%, respectively). Disturbance during daily business activities (14%) and cold sensitivity (23%) were significantly lower in Group 1 compared to Group 2 (86% and 77%, respectively).

**Conclusions:**

Semi-occlusive dressing therapy for fingertip amputations demonstrated excellent healing rates. Compared to surgical treatment, it resulted in significantly better clinical outcomes, lower complication rates and significantly higher reported satisfaction rates. Therefore, semi-occlusive dressing for fingertip injuries is a very successful procedure and shall be preferred over surgical treatment in most cases.

**Level of evidence:**

III therapeutic.

## Introduction

Fingertip injuries are among the most common hand injuries being the reason for approximately 4.8 million emergency visits per year [1]. The main treatment aim is to achieve a functional, stable and cosmetically appealing coverage of the fingertip. However, treatment strategies vary depending on not only the degree of tissue loss and site of injury but also on the cultural diversity of involved patients [2–5].

Available treatment options range from semi-occlusive dressings to a broad range of surgical techniques. Surgical techniques include local, regional and even distant flaps as well as replantation or composite grafting [6]. The selected technique not only depends on the surgeon`s skills, but also on patient’s comorbidities and the extent of tissue injury. Despite numerous advances in the treatment of these injuries, there is still no consensus on how they should be managed. In the recent years, conservative treatment using semi-occlusive dressings has become popular with excellent results reported in numerous studies [2, 7–12]. Moreover, the indication for semi-occlusive dressing therapy has been extended to fingertip amputations with exposed bone showing good clinical and aesthetic outcomes [13].

Although there have been several studies on conservative and surgical treatment of fingertip amputations, a direct comparison between surgical treatment and semi-occlusive dressing has not been investigated so far.

It was therefore the aim of this study to (1) analyse healing rates of occlusive dressing therapy and surgical treatment for fingertip amputations, (2) assess complications of the procedures, and (3) compare the two treatment strategies in terms of clinical outcome and patient satisfaction.

## Materials and methods

### Ethical approval

Ethical approval for this retrospective clinical study was granted by the responsible institutional review board (BASEC 2017-01471) and all included patients provided written consent.

### Patients

A retrospective chart review was performed and patients with amputated fingertips treated with either semi-occlusive dressings or surgery at our institution between March 2012 and July 2017 were identified. Patients eligible for inclusion were at least 18 years old. Severe additional hand injuries, prior major hand surgery and distal interphalangeal joint (DIP) affection were defined as exclusion criteria. In a first step, 107 patients were identified. Following application of the exclusion criteria, 84 patients (79%, 14 women, 70 men) with a mean age of 45 years (range 19–80 years) at the time of treatment were allocated for further clinical examination with a mean follow-up of 28.1 months (range 9.6–46.2 months). Patients treated with semi-occlusive dressing were assigned in Group 1 and patients treated surgically in Group 2. Since 2016, occlusive dressing is the preferred therapy for fingertip amputations at our institution. Therefore, we identified the last 18 patients (21%) treated with surgery by the end of 2015. Of these 18 patients 6 (33%) received a stump formation with primary wound closure, 4 (22%) a replantation of the fingertip, 3 (17%) a composite graft, 3 a cutaneous flap and 2 (11%) a neurovascular flap. Because the respective numbers of individual surgeries were too low for further analysis, we combined them all into one surgical group (Group 2) and compared them with the remaining 66 patients (79%), who were treated conservatively with semi-occlusive dressings (Group 1). However, it is worth mentioning that the bone of the distal phalanx was shortened back to the level of soft tissue injury if exceeding the level of injury. The nail was left untouched in all patients. No significant differences were detected between the groups with regard to the anthropometrics data of included patients (Table 1).

**Table 1:** Detailed anthropometrics of included patients

Variable	Group 1 (*n* = 66)	Group 2 (*n* = 18)	*p* value
Age (years)	45.8 (range 18.5–79.9)	42.2 (range 22.5–58.5)	0.40
Sex	54♂, 12♀	16♂, 2 ♀	0.48
Smokers, non-smokers (*n*)	17, 49	7, 11	0.27
Pack years	27.1 (range 10–45)	26.4 (range 10–40)	0.91
Manual labourer *n*, (%)	41 (62.1)	15 (83.3)	0.09
Dominant side affected *n*, (%)	31 (47.0)	5 (27.8)	0.14
Follow up (months)	24.1 (range 3.4–46.4)	28.1 (range 9.6–46.2)	0.48
Affected finger^+^ (*n*)	1 = 16; 2 = 19; 3 = 15; 4 = 10; 5 = 6	1 = 3; 2 = 9; 3 = 4; 4 = 1; 5 = 1	0.47
Trauma mechanism* (*n*)	1 = 19; 2 = 29; 3 = 18	1 = 3; 2 = 10; 3 = 5	0.54
Allen Classification (1–4)	1.7 (1–4); median = 2	2.2 (1–4); median = 2	0.24
Amputated fingertip in (%)	43 (range 15–70)	47 (range 15–70)	0.26
Affected side^$^	1 = 19; 2 = 23; 3 = 24	1 = 8; 2 = 5; 3 = 5	0.45

*y/n* yes/no

^+^Affected finger: 1 = thumb, 2 = index finger, 3 = middle finger, 4 = ring finger 5 = little finger; *Trauma mechanism: 1 = sharp, 2 = laceration, 3 = avulsion; ^$^1 = palmar, 2 = dorsal, 3 = equally affected

### Clinical assessment

Clinical outcome parameters included pain, lost working days and complications. Clinical examination included assessment of active ranges of motion measurement of affected metacarpophalangeal (MCP), proximal interphalangeal (PIP) and DIP joints as well as their uninjured contralateral counterparts. Furthermore, trophic finger or nail changes were rated (yes/no). Jamar^®^ hydraulic hand dynamometer (J. A. Preston Corporation, Clifton, NJ, USA) [14] was used for measuring grip strength in both hands of each participant in an upright straight sitting position, 90° flexion of the elbow and handle position at level 2 for all measurements. Furthermore, two-point discrimination of the injured and contralateral fingers was measured using a Dellon Disk-Criminator (NexGen Ergonomics Inc., Quebec, Canada) [15]. Patients were assessed using the quick disabilities of arm, shoulder and hand score (Quick-DASH) [16], which is a further development of the DASH score with a limit of 11 questions. When interpreting the results of this score, one must keep in mind that lower values reflect a better clinical result. Patients were additionally accessed using the visual analog scale (VAS) [17] at rest and after exposure. Furthermore, patients rated their subjective aesthetic outcome on a scale from 0 to 100, reported disturbances during daily business activities (yes/no), and indicated whether they would go through the same treatment again (yes/no). Cold sensitivity (yes/no), numbness (yes/no) and electrical sensations (yes/no) were reported for the affected fingertips. All fingertip amputations were rated by the first author according to Allen Classification [18], according to site (“palmar”, “dorsal” or “equal”) and to lost percentage of the end phalanx. The Allen Classification was first described in 1980 and distinguishes between four types:

- Type I: only the pulp of the finger is involved; distal to nail bed.
- Type II: distal to distal phalanx; pulp and nail loss.
- Type III: distal to lunula; loss of terminal phalanx additionally to loss of pulp and nail.
- Type IV: distal to DIP-Joint, lunula of the nail and pulp are involved as well as nail and partial loss of the terminal phalanx.

### Statistical analysis

Statistical analysis was performed with SPSS software package (IBM SPSS Statistics, V27, IBM, Armonk, NY, USA). Shapiro–Wilk test was used to screen normality of data distribution. Mann–Whitney-*U* and *χ*^2^ tests were applied to detect significant differences between the groups. Level of significance was set to 0.05 for all statistical tests.

## Results

### Complications and re-interventions

After a mean of 17 (range 2–38) days, 5 of the 18 surgically treated patients (28%) were with necrosis of the fingertip necessitating a change in treatment strategy. After a semi-occlusive dressing was applied, all these 5 patients ultimately healed uneventfully (see Table 2 for individual description of surgical complications).

**Table 2:** Individual description of surgical complications in Group 2 for 5 out of 18 patients (28%)

Patient	Sex	Age at surgery	Surgery	Days to failure	Complication	Smoking	Ultimately healed
1	♀	22	K-wire osteosynthesis, composite graft and nailbed suture	38	Graft necrosis	No	Yes
2	♂	59	Primary wound closure with nail eradication	2	Impending necrosis	Yes	Yes
3	♂	38	Cutaneous flap	3	Flap necrosis	No	Yes
4	♂	43	Replantation	30	Necrosis	Yes	Yes
5	♂	38	Composite graft*	14	Graft necrosis	Yes	Yes

*External surgery with secondary referral to our institution

Fingertips healed uneventfully in all 66 conservatively treated patients within 6 weeks. However, minor complications occurred such as cold sensitivity, numbness, electrical sensations, trophic changes of fingertip and nail, loss of 2-point-discrimination as well as loss of flexion in the finger joints (see Table 3 for detailed description). Nail deformations found in the surgical group were split nail deformity, scarring of the nail bed, nail remnants and undulating nail growth. In the conservative group nail remnants and undulating nail growth were considered as nail deformities.

**Table 3:** Clinical findings at final follow-up

Variable	Group 1	Group 2	Difference	*p* value
VAS at rest (pts)^$^	0.0 ± 0.0	0.3 ± 1.0	0.3	0.31
VAS at exposure (pts)^$^	0.2 ± 1.0	1.4 ± 2.0	1.2	< 0.01*
Quick-DASH absolute (pts)^$^	11.4 ± 1.0	12.8 ± 5.0	1.4	0.06
Quick-DASH value (%)^$^	0.9 ± 2.0	4.0 ± 12.0	3.1	0.06
Subjective aesthetics (%)^$^	86.7 ± 17.0	77.2 ± 17.0	− 9.5	0.01*
Working days lost^€^	28.2 (1–98)	61.7 (14–140)	33.5	< 0.01*
Repeat same treatment *n*, (%)	63 (95.5)	16 (88.9)	− 6.6	0.30
Cold sensitivity *n*, (%)	15 (22.7)	9 (44.4)	33.9	0.02*
Numbness *n*, (%)	2 (3.0)	0 (0.0)	− 3.0	0.45
Electrical sensations *n*, (%)	2 (3.0)	2 (11.1)	8.1	0.15
Disturbance daily business *n*, (%)	9 (13.6)	8 (44.4)	30.8	< 0.01*
Trophic changes finger *n*, (%)	21 (31.8)	13 (72.2)	29.5	< 0.01*
Trophic changes nail *n*, (%)	12 (18.2)	9 (50.0)	19.7	< 0.01*
Loss of MCP flexion (°)^+ $^	1.7 ± 5.0	2.2 ± 4.0	0.5	0.17
Loss of MCP extension (°)^+ $^	0.0 ± 0.0	0.0 ± 0.0	0.0	1.00
Loss of PIP flexion (°)^+ $^	1.2 ± 3.0	2.6 ± 3.0	1.4	0.06
Loss of PIP extension (°)^+ $^	0.0 ± 0.0	0.0 ± 0.0	0.0	1.00
Loss of DIP flexion (°)^+ $^	3.8 ± 5.0	10.6 ± 19	6.8	0.45
Loss of DIP extension (°)^+ $^	0.0 ± 0.0	0.0 ± 0.0	0.0	1.00
Loss of Grip strengths (kg)^+ $^	2.9 ± 6.0	3.9 ± 9.0	1.0	0.99
Loss of 2-point-discrimination (mm)^+ €^	1.0 (0–4)	1.9 (0–5)	0.9	0.03*

*Pts* points, *VAS* visual analogue scale, *MCP* metacarpophalangeal joint, *PIP* proximal interphalangeal joint, *DIP* distal interphalangeal joint, *y/n* yes or no

^$^Data are presented as mean ± standard deviation; ^+^compared to the healthy side; ^€^data are presented as mean (range); *statistically significant

### Clinical outcome

**Fig. 1:**
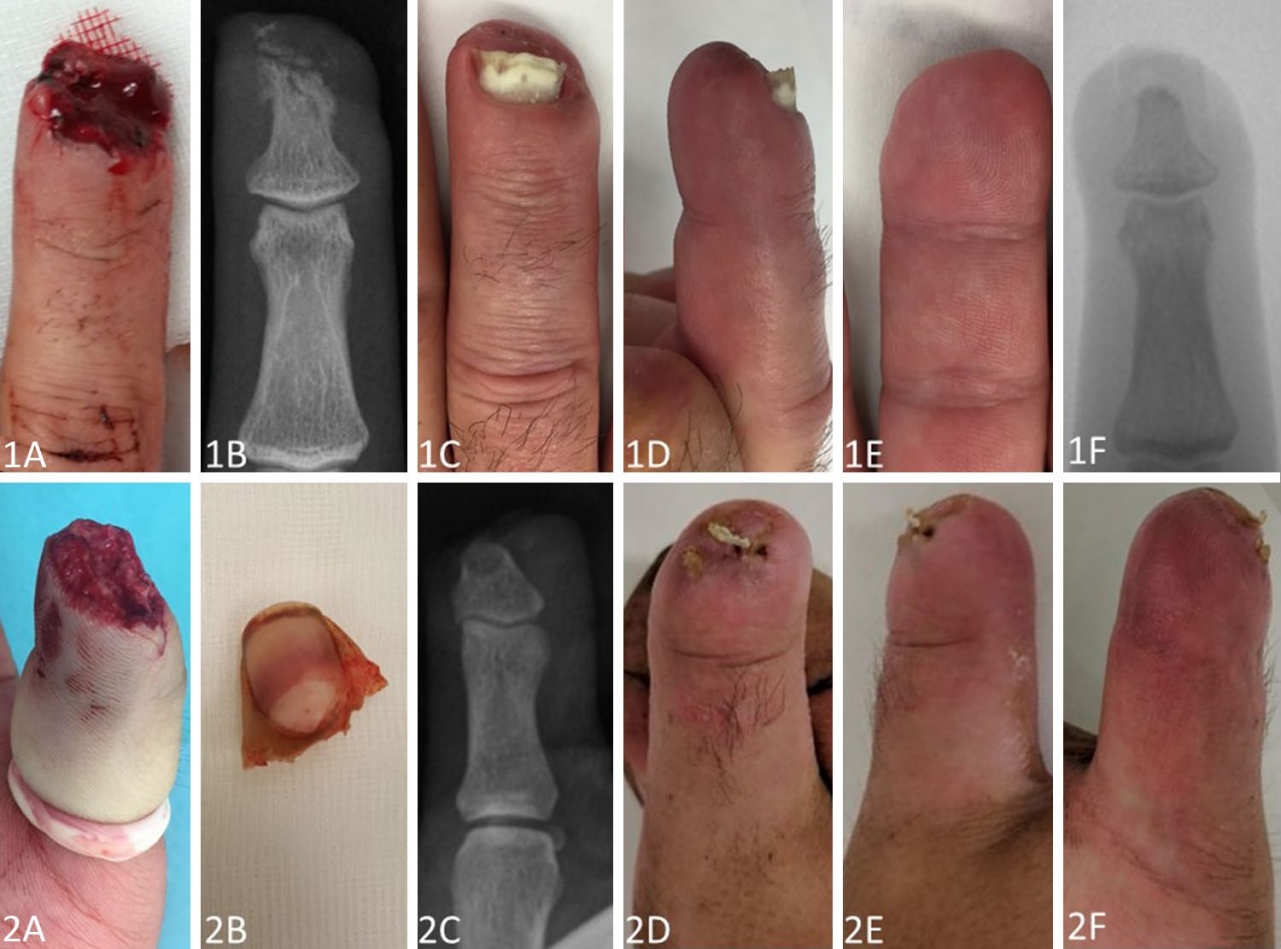
**Case 1 (1A-F)**: pictures taken after a milling accident of the right digit 4 of a 30-year-old farmer. Considering the nail matrix laceration (**1A)** and affected bone (**1B)**, this finger was rated with grade 3 according to Allen Classification. **1C-F**: result after semi-occlusive dressing therapy at a follow-up of 2.5 months. Note: minor trophic changes of the nail. **Case 2 (2A-F)**: pictures taken after a planning injury of the right thumb of a 22-year-old lumberjack. Considering the amputation level being proximally to the lunula of the nail **(2A-C)**, this finger was rated with grade 4 according to Allen Classification. **2D-F**: Result after semi-occlusive dressing therapy at a follow-up of 1.5 months. Note: trophic changes of the nail

**Fig. 2:**
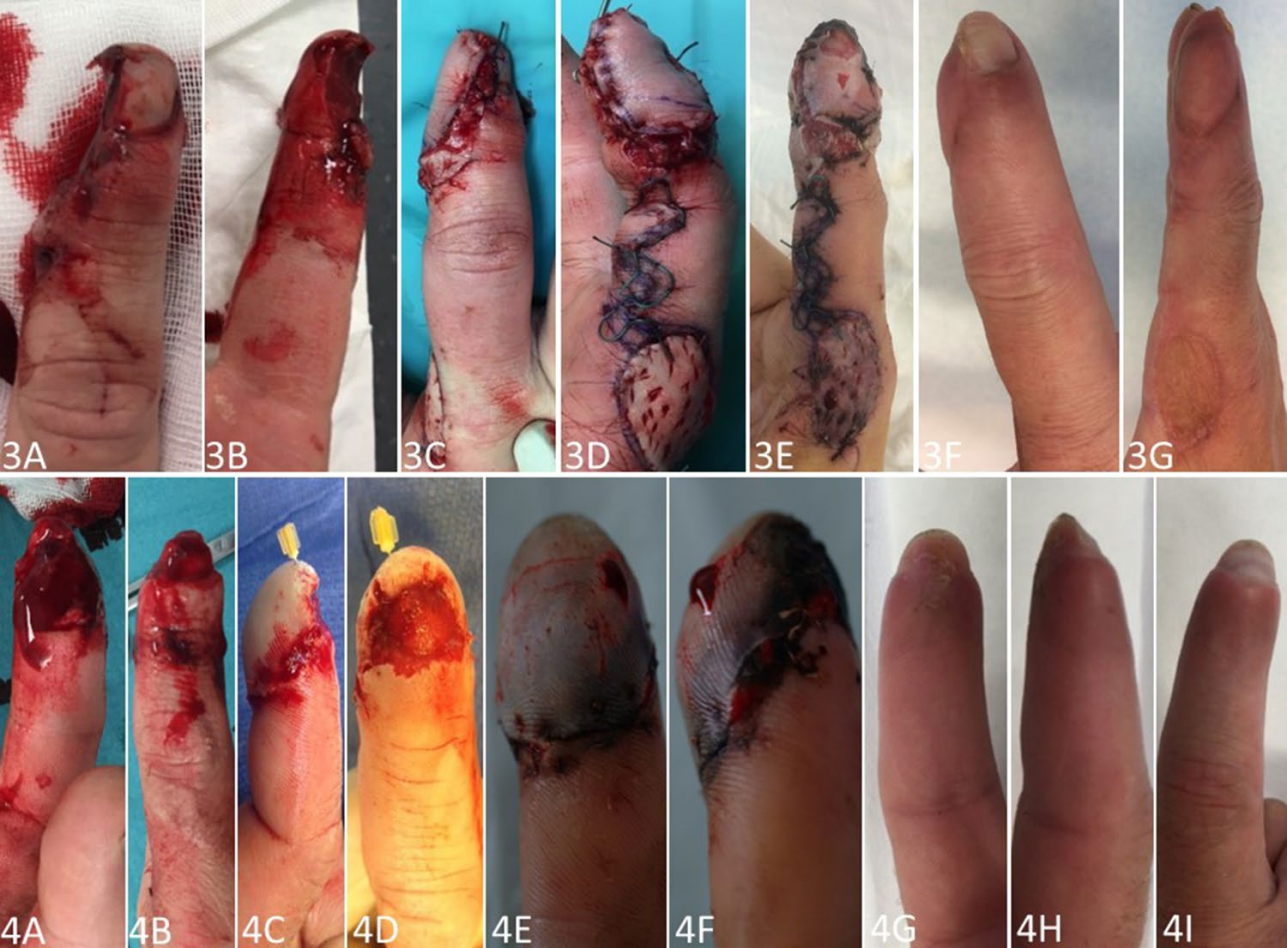
**Case 3 (3A-G)**: pictures taken after a milling accident of the right index finger of a 50-year-old carpenter. Considering the partial nail matrix laceration **(3A)** and affected bone **(3B)**, this finger was rated with grade 3 according to Allen Classification. Result after a homodigital island flap treatment post operation **(3C-E)** and at a follow-up of 2 years **(3F, G)**. Note: minor trophic changes of the nail and pulp as well as the donor side aesthetics. **Case 4 (4A-G)**: pictures taken after a rolling injury of the right index finger of a 43-year-old pharmaceutical specialist. Considering the amputation level distally to the lunula of the nail **(4A, B)**, this finger was rated with grade 2 according to Allen Classification. **4C, D**: result after replantation of the pulp post operation **4E, F**: due to necrosis after 5 days treatment was changed to semi-occlusive dressing therapy after debridement. **4G-I**: result after 3 months. Note: atrophic changes of the pulp and nail

Eight out of the 84 patients were already retired, thus leaving 76 working patients at time of the accident, of which all 100% were able to return to their previous occupation after a mean of 62 (range 14–140) days. However, patients receiving a semi-occlusive dressing were able to return significantly earlier to work (*p* < 0.01). Furthermore, patients treated conservatively with a semi-occlusive dressing demonstrated significantly lower VAS scores during exposure (*p* < 0.01), reported significantly better subjective aesthetics (*p* = 0.01), significantly less cold sensitivity (*p* = 0.02) and reported significantly less disturbances during daily business activities (*p* < 0.01). Moreover, conservatively treated patients demonstrated significantly less trophic changes of the finger (*p* < 0.01) and nail (*p* < 0.01) as well as a significantly reduced loss of 2-point-discrimination as compared to surgically treated patients (*p* = 0.03). Clinical findings at the final follow-up are presented in Table 3 with no further significant differences between the groups. However, Quick DASH scores and loss of PIP flexion were lower in group 1 with a strong trend to significance (*p* = 0.06). Two exemplary cases for patients treated with semi-occlusive dressings are presented in Fig. 1 and two cases representing operative treatment are depicted in Fig. 2.

## Discussion

The main findings of the current study demonstrate: (1) a 100% wound healing rate of fingertip amputations treated conservatively with a semi-occlusive dressing therapy in contrast to a complication rate of 28% in surgically treated patients; (2) reasons for failure in the surgical group of patients were graft necrosis in all cases; (3) patients treated conservatively demonstrated significantly lower pain at exposure, less cold sensitivity, less disturbance during daily business activities, less trophic changes in fingers and nails, a better 2-point discrimination and were able to return earlier to work. Although there have been numerous studies on conservative and surgical treatment of fingertip amputations, this study is the first to directly compare both treatment options for fingertip amputations.

Treatment aims in fingertip reconstruction are an adequate soft tissue coverage, maximized sensory rehabilitation, preserved length of the digit, maintained joint movement, an obtained satisfactory cosmetic appearance and patient satisfaction. The results of the current study in terms of excellent healing rates for semi-occlusive dressing therapy are in line with previous reports [2, 7–12]. In 1974, Illingworth described her first young patient suffering a fingertip amputation with wound covered by a simple dressing [19]. The child was seen at the plastic surgery department due to a misunderstanding several days after the initial consultation at the emergency department. After this short period of time the young patient presented a very satisfying wound healing and eventually a regrowth of the fingertip. Douglas reported the same findings in Australia in 1972 [20] stating that fingertip injuries may heal satisfactorily without surgical treatment.

Besides, in 1975 Bossley performed a study on 55 patients showing good healing potential using Vaseline gauze [21]. In 1993 Mennen and Wiese were the first ones describing semi-occlusive dressing therapy with very good clinical results in 200 cases [11]. Despite these promising results, semi-occlusive dressing had not become established for many years. However, it has been experiencing a resurgence in the recent years, mainly in Europe [22–24]. Other parts of the world, especially Asian countries, seem to be more aggressive with the surgical procedures such as local flaps treatment resulting in acceptable healing rates [3–5]. The VY flap, first described in 1935 by Tranquilli-Leali, is still one of the most used flaps in modified ways [25]. Nevertheless, this local flap often leads to a reduced soft tissue cushion with a narrowed and flattened fingertip [24]. For a long period of time surgeons believed that semi-occlusive dressing therapy would lead to a flattened and reduced soft tissue cushion at the fingertip too. However, Hoigné et al. demonstrated high regeneration potential of the soft tissue thickness applying ultrasound methods [13]. They could further demonstrate a 90% restoration of the soft tissues compared to the contralateral healthy side. The patients in the current study who suffered an amputation at grade 4 according to Allen Classification regained an adequate soft tissue coverage and restoration of the pulp, which might be a major problem after surgical treatment. (See Fig. 1: 1D, E and 2E, F in contrast to Fig. 2: 3F, G and 4G, H).

Disadvantages of this therapy are regular (usually once a week) consultations at the outpatient clinic for a period of at least 3–4 weeks [26]. Moreover, odor and liquid under the dressing are found to be bothersome by some patients. However, semi-occlusive dressing treatment demonstrated significantly lower VAS scores, lower loss of 2-point-discrimination, lower trophic changes in finger and nail, less disturbance in daily business activities and less cold sensitivity. Furthermore, there was a strong trend to a lower Quick DASH score and lower loss of PIP flexion in the semi-occlusive-dressing group compared to the surgical group. These findings are in line with the work of Söderberg et al. who demonstrated restricted movements in DIP joints, infections, chronic pain, hypersensitivity to cold, paraesthesias as well as less sensitivity, all leading to an impairment in daily activities for patients with operatively treated fingertips [27]. In surgically treated fingertip amputations postoperative healing is not always without concerns. Quite often surgeons are faced with wound healing deficits such as flap necrosis or infection. In the current study flap necrosis was observed in 28% of the cases which led to a change of the therapy regime. This finding is consistent with previous reports [24, 27]. The relatively high rate of flap necrosis in the surgery group might be due to the poor condition of the vessels after a traumatizing injury. This is the main reason why composite grafts often do not result in healing after crushing injuries [26]. Another aspect is that regional flaps such as the VY-flap described by Kutler [28], or the Segmüller [29] and Venkataswami flap [30] must be well mobilized in order to create not too much tension at the distal wound. While mobilizing the flap, attention must be paid to incise only through the dermis and preserve arborizing vessels [28, 30].

Wounds deeper than the dermis heal with scar tissue. Nevertheless, fingertip wounds are able to heal by secondary intentions without producing a scar [13]. This might be due to inhibition of fibroblast proliferation under the influence of the secretion [31]. Although there have been several studies on amphibians, reptiles and mice, the regeneration processes in human fingertips are still not fully understood [23, 32, 33].

The current study demonstrates a significantly shorter work absence in the group of the semi-occlusive dressing. On average, return to work was 33.5 days earlier than in the surgically treated group. Wound healing in the surgical group needed a longer after care and change of treatment in 28% of the cases. There were no wound healing issues in the semi-occlusive-dressing group—with no influence of patient comorbidities. Martin and del Pino reported that surgically treated patients returned to work after 1–2 months, which is in line with the results of the current study [26]. Due to the promising results in the literature, semi-occlusive dressing therapy is gaining more clinical and tissue engineering research attention. At our institution we prefer surgical options in situations with exposed flexor or extensor tendons or large portions of exposed bone [22], when shortening of the digit is no option.

Limitations of this study include the retrospective design with prospective follow-up and only 18 surgically treated patients. In addition, a pinch gauge to compare the strength each finger of the injured hand with its contralateral counterpart was not used in the current study. Furthermore, patients in the surgical group were treated with different operation types; thus conclusions may vary for individual surgeries. Larger patient collectives are necessary for detailed subgroup analysis. The systematic collection of complete clinical data for all patients undergoing surgery for fingertip amputations in our institution and a follow-up rate of 79% is, however, a robust basis for the present study.

## Data Availability

All data relevant to the study are included in the article or uploaded as supplementary information.
